# Iatrogenesis in mental health care

**DOI:** 10.1192/j.eurpsy.2021.2045

**Published:** 2021-08-13

**Authors:** M. Sánchez Revuelta, J. Matsuura, C. Martín Villarroel, L. Carpio Garcia, J. Dominguez Cutanda

**Affiliations:** Psychiatry, COMPLEJO HOSPITALARIO DE TOLEDO, Toledo, Spain

**Keywords:** iatrogenesis, schizophrénia, Psychotropic drugs, side effects

## Abstract

**Introduction:**

The need for preventive mechanisms in psychiatric pathology has been raised, therefore authors talk about primary, secondary and tertiary prevention. However, this emphasis on those preventive aspects has tended to ignore an essential part: quaternary prevention.

**Objectives:**

Reflecting the importance of avoiding ignoring iatrogenic forms of psychopathology by studying a clinical case and reviewing available literature.

**Methods:**

We will present a clinical case of a patient with residual schizophrenia who undergoes an escalation of pharmacological interventions that lead to functional deterioration after initiating behavioral alterations. We will also review available literature about quaternary prevention.

**Results:**

M. is an institutionalized patient who was taking a combination of three neuroleptics, anxiolytics and stabilizers for the treatment of behavior problems such as heteroaggressiveness. When the patient was referred to psychiatry consultations after being hospitalized, he could not move, had lost sphincter control and had serious communication problems. However, treatment was suspended and only one neuroleptic was maintained. The patient regained sphincter control and kept a residual but communicative delusional speech.

**Conclusions:**

It is important to see how sometimes we can get into therapeutic escalation without correcting the underlying problem by focusing on a symptom, because behavioral alterations will persist regardless of pharmacological treatment changes. Sometimes clinical fluctuations make us confuse basal state and decompensation, ignoring the fact that we lack the way to modify the course. Authors believe that a rational approach to treatment should take into account the balance between potential benefits and side effects applied to an individual patient.

**Disclosure:**

No significant relationships.

